# A Systematic Review of the Advances in the Study of T Lymphocyte Suppressor Receptors in HBV Infection: Potential Therapeutic Targets

**DOI:** 10.3390/jcm13051210

**Published:** 2024-02-21

**Authors:** Daqiong Zhou, Lili Liu, Jiangyu Liu, Hong Li, Jing Zhang, Zhenhuan Cao

**Affiliations:** The Third Unit, Department of Hepatology, Beijing Youan Hospital, Capital Medical University, Beijing 100069, China; zhoudq2022@163.com (D.Z.); liulilidoc@126.com (L.L.); liujiangyu@mail.ccmu.edu.cn (J.L.); lihong199612@163.com (H.L.)

**Keywords:** hepatitis B virus, chronic HBV infection, immune checkpoints, inhibitory receptors, T cell exhaustion

## Abstract

**Background:** HBV-specific T lymphocytes are pivotal in eliminating the hepatitis B virus (HBV) and regulating intrahepatic inflammatory reactions. Effective T cell responses curtail HBV infection; however, compromised immunity can result in persistent infection. Beyond the acute phase, the continued presence of antigens and inflammation leads to the increased expression of various inhibitory receptors, such as PD-1, CTLA-4, Tim-3, LAG3, 2B4, CD160, BTLA, and TIGIT. This escalates the dysfunction of and diminishes the immune and proliferative abilities of T cells. **Methods:** In this study, we reviewed English-language literature from PubMed, Web of Science, and Scopus up to 9 July 2023. This paper aims to elucidate the inhibitory effects of these receptors on HBV-specific T lymphocytes and how immune function can be rejuvenated by obstructing the inhibitory receptor signaling pathway in chronic HBV patients. We also summarize the latest insights into related anti-HBV immunotherapy. **Result:** From 66 reviewed reports, we deduced that immunotherapy targeting inhibitory receptors on T cells is a reliable method to rejuvenate T cell immune responses in chronic HBV patients. However, comprehensive combination therapy strategies are essential for a functional cure. **Conclusions:** Targeting T cell suppressor receptors and combining immunotherapy with antiviral treatments may offer a promising approach towards achieving a functional cure, urging future research to prioritize effective combination therapeutic strategies for chronic HBV infection.

## 1. Introduction

Hepatitis B virus (HBV) infection remains a significant global health challenge, affecting approximately 296 million individuals globally. Annually, 2% to 5.5% of HBeAg-positive chronic hepatitis B (CHB) patients progress to cirrhosis, with a 5-year progression rate of 8% to 20%. In contrast, HBeAg-negative CHB patients advance to cirrhosis at a more rapid pace of 8% to 20% annually. Notably, between 25% and 40% of HBV carriers are at risk of developing liver cancer [1,2]. Given these statistics, devising effective strategies to eradicate the virus and prevent liver failure, cirrhosis, and liver cancer is of paramount importance. HBV-specific T cells play a pivotal role in the progression of the disease, impacting factors such as viral clearance and the onset of hepatitis, cirrhosis, and liver cancer. They also significantly influence the response to antiviral treatment [3].

T lymphocyte activation necessitates two signals: the first stems from MHC molecules presenting antigens to T cells, while the second arises from the binding of receptors on T cells to ligands on antigen-presenting cells (APCs). This receptor–ligand pairing is termed an immune checkpoint [4]. T cells express two sets of costimulatory receptors. One set transmits stimulatory signals related to naïve T cell activation, while the other functions as checkpoints for T cell activation, ensuring immune tolerance and homeostasis during infections. Chronic HBV infections illustrate how, as the infection persists, T cells tend to become dysfunctional. This is marked by a reduced secretion of cytotoxic factors like IL-2, IFN-γ, and TNF, paired with a heightened release of immunosuppressive agents such as IL-10 and TGF-β. Additionally, inhibitory receptors become more prominent [5,6].

One hypothesis suggests that the inability to effectively eliminate HBV might stem from inhibitory receptors impairing T lymphocyte function. This review systematically delineates the immunoregulatory functions of T lymphocyte-associated inhibitory receptors in the context of hepatitis B virus (HBV) infection and compiles data from clinical trials where these receptors have been leveraged in the immunotherapeutic management of HBV infection (please consult Table 1). Drawing upon contemporary foundational research and clinical developments, this paper offers novel perspectives and potential future trajectories for research into inhibitory receptors within the realm of HBV infection.

## 2. Materials and Methods

This systematic review adhered to the 2020 Preferred Reporting Items for Systematic Evaluation and Meta-Analysis (PRISMA) guidelines [7]. A flowchart for report screening was constructed in alignment with the associated criteria and specifications.

### 2.1. Eligibility Criteria

This study incorporated records based on the following criteria: (1) research involving subjects related to HBV infection, encompassing HBV-infected patients, in vitro HBV cellular models, and animal models of HBV infection; (2) clinical conditions exclusively linked to HBV infections, such as AHB, CHB, HBV-HCC, and HBV-ACLF; (3) a focus on T cells and their respective subgroups; (4) articles published in English; and (5) original articles or abstracts containing complete data.

### 2.2. Exclusion Criteria

Studies were excluded for the following reasons: (1) they integrated infections from other viruses like HIV, HCV, HDV, influenza virus, and CMV; (2) they were reviews, letters, case reports, and book chapters; (3) they combined HBV infections with other malignancies; (4) they investigated hepatocellular carcinoma of unidentified origins or those attributed to different causes; and (5) they focused on inhibitory receptors expressed in other immune cells, including DCs, NK, NKT, and B cells.

### 2.3. Data Sources and Search Strategy

This study sourced reports from the literature available up to 22 July 2023, within the following databases: PubMed, Web of Science, and Scopus. A systematic literature search employed the following keywords: “hepatitis B” OR “HBV”, “inhibitory receptors” OR “inhibitory checkpoints” OR “immunization checkpoints”, and “T lymphocyte” OR “T cell”. Details of the specific search procedure and its results can be found in Table 2.

### 2.4. Record Screening Process

Using EndNote 20 software (Clarivate Analytics, London, UK), the acquired documents were screened and filtered based on the predefined inclusion and exclusion criteria. Only the records that aligned with the study’s requirements were ultimately chosen.

### 2.5. Synthesis of Results

This study primarily centered on human research, while other investigations, such as animal and in vitro studies, were referenced as corroborative evidence to support the main outcomes observed.

## 3. Results

**Figure 1:**
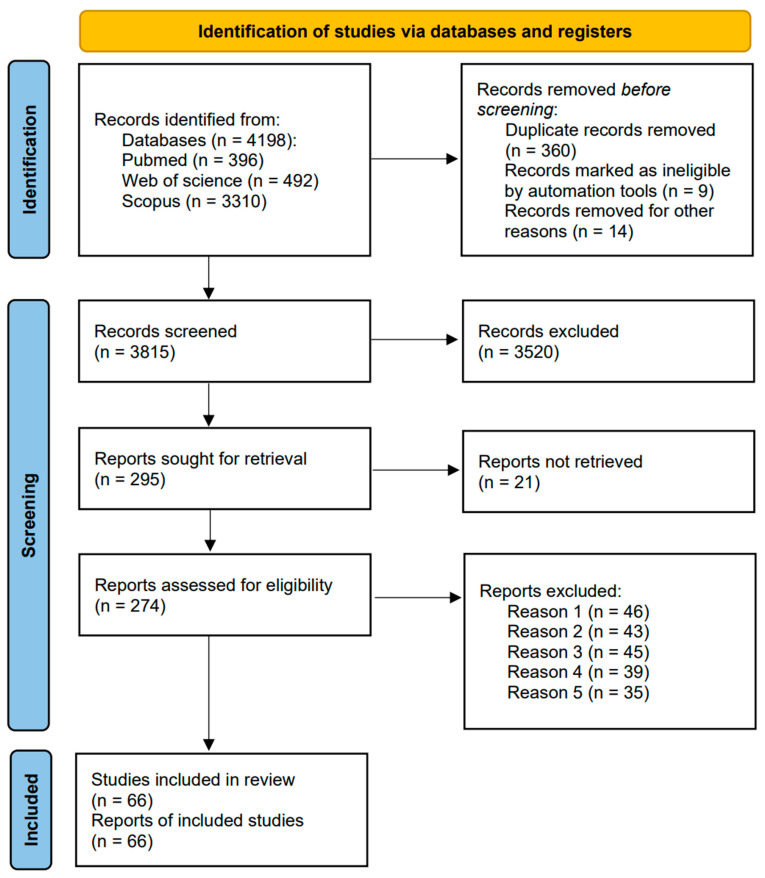
PRISMA 2020 flow diagram of the studies included in the systematic review. Reason 1: review; reason 2: non-compliant results; reason 3: research target is HCC; reason 4: other viral infections; reason 5: associated with other cells.

Based on the specified search terms, we identified 4198 records from the databases as follows: Pubmed (396), Web of Science (492), and Scopus (3310). After applying automated screening conditions and the inclusion criteria, 66 studies were shortlisted for review. Please refer to Figure 1 for the specific screening process.

### 3.1. Programmed Cell Death-1 (PD-1)

#### 3.1.1. PD-1 Expression on Normal T Cells

PD-1 belongs to the CD28/CTLA-4/ICOS immunoglobulin transmembrane protein superfamily, playing a critical role in modulating T cell function throughout various stages of T cell differentiation [8]. During the early stages of T cell activation, PD-1 engages with its ligands, PD-L1 (B7-H1; CD274) or PD-L2 (B7-DC; CD273), initiating inhibitory signaling pathways that dampen T cell activation signals. This regulatory mechanism affects T cell metabolism, decreasing IL-2 production and promoting IL-10 secretion. As a result, T cell proliferation and functional response are inhibited [9,10,11]. The cumulative impact of the PD-1 signaling pathway is a comprehensive inhibition of the immune response in effector T cells, evident in diminished proliferation, reduced effector cytokine synthesis, and decreased cytotoxicity. Furthermore, the expression of PD-1 on regulatory T (Treg) cells, known for their immunosuppressive capabilities, exerts a vital regulatory influence on the immune functions of effector T cells. Specifically, the presence of PD-1 on Treg cells correlates with a reduction in their suppressive activity. This phenomenon underscores the significance of the balance between PD-1+ CD8+ T cells and PD-1+ Treg cells in determining the overall immune response of the organism [12].

#### 3.1.2. PD-1 Expression on HBV-Specific T Cells

**Figure 2:**
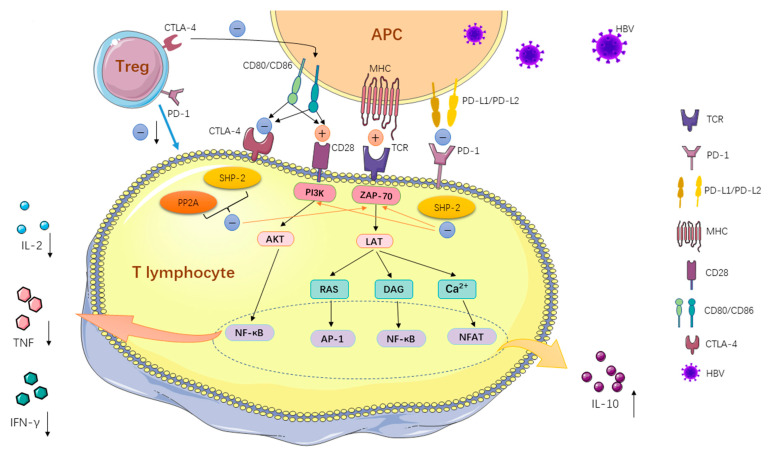
As shown, binding of PD-1 to its two ligands activates inhibitory signaling pathways that induce phosphorylation of tyrosine residues on the PD-1 cytoplasmic structural domain upon antigen stimulation, leading to recruitment of SHP-2 to attenuate T cell activation signals and modulate metabolic reprogramming during initial T cell activation, inhibiting the TCR and CD28 signaling-driven upregulation of glucose and upregulation of glutamine metabolism, thereby antagonizing cell activation stimulatory signals transduced through the TCR and CD28 pathways and thereby inhibiting T lymphocyte function. Antigen stimulation of the organism induces T lymphocytes to overexpress CTLA-4 and recruit large amounts of the phosphatases SHP2 and PP2A into the cytoplasm, thereby inhibiting TCR signaling and leading to a decrease in both T cell proliferation and cytokine production. Alternatively, PD-1 and CTLA-4, expressed on Treg cells, can regulate the immune function of T cells directly or indirectly by regulating the expression of inhibitory ligands on APCs. “↑” represents an increase in quantity or magnitude, while “↓” represents a decrease. (Created with https://smart.servier.com/, accessed on 12 June 2023).

In chronic hepatitis B patients, PD-1 is predominantly observed on lymphocytes near the portal vein, while PD-L1 is expressed on lymphocytes, hepatocytes, and hepatic sinusoidal endothelial cells. PD-L2 is found on Kupffer cells and dendritic cells. Studies suggest that the expression of PD-1 and PD-L on lymphocytes and antigen-presenting cells (APCs) may contribute to local immune dysfunction and the persistence of HBV infection [13] (Figure 2). The dynamics of PD-1 expression on T lymphocytes are closely linked to the disease state and the severity of inflammation in HBV infections. During the initial phases of acute HBV infection, there is a significant increase in PD-1 expression on HBV-specific CD8+ T cells. This elevated PD-1 expression dampens CD8+ T cell function, regulating CD8+ T cell responses and mitigating liver damage. Conversely, if PD-1 expression on HBV-specific CD8+ T cells is delayed, it may contribute to acute liver failure [14]. Similar findings were observed in patients with acute exacerbation of HBV infection (AEHB), where CD8+ T lymphocytes exhibited lower levels of PD-1 compared to those with chronic HBV infection. Moreover, AEHB patients showed a stronger HBV-specific CD8+ T cell response compared to chronic HBV patients [15]. These findings suggest that a moderate increase in lymphocyte PD-1 expression may serve as a host defense mechanism against harmful immune responses. In the context of HBV infections, the interactions between PD-1 and its ligands in the liver might be crucial for balancing immune responses aimed at clearing HBV infection with those causing immune-mediated liver damage.

#### 3.1.3. Blocking the PD-1/PD-L1 Pathway in the Treatment of HBV Infection

Multiple studies have validated the safety and efficacy of anti-PD-1 blockers in treating CHB patients. Notably, checkpoint blockade therapy was well received in HBeAg-negative patients with viral counts below detection limits, observing varying degrees of HBsAg reduction in the majority of these patients [16]. Nonetheless, the capacity of anti-PD-1 blockers to revitalize depleted HBV-specific T lymphocytes seems restricted. PD-1 blockade was found to augment T cell function in patients with minimal HBV-specific T lymphocyte depletion, but the response was less pronounced in patients with extensive T lymphocyte depletion [17]. This hints at the potential limited clinical effectiveness of solely relying on the PD-1/PD-L1 blockade and underscores the need for integrated therapeutic strategies. Some studies have reported the synergistic impact of combining PD-1 blockade and anti-HBV therapy. This combination not only suppressed HBV replication but also notably improved the survival rates of HBV transgenic mice. Additionally, this dual therapy fostered the revival of T cell function, increased IFN secretion, and reshaped the HBV infection’s immune microenvironment [18,19].

More than 3300 clinical studies involving anti-PD-1 monoclonal antibodies have been conducted across various conditions and diseases, ranging from solid tumors to infectious diseases. Currently, seven studies linked to anti-PD-1 monoclonal antibodies and HBV infection are registered on the National Library of Medicine’s Clinical Trials Platform (https://clinicaltrials.gov, accessed on 9 July 2023). Of these, two, NCT05771402 and NCT05769816, have progressed to phase IV clinical trials. While these studies aim to explore the efficacy and safety of combining anti-PD-1 monoclonal antibodies with NAs and IFN-α, respectively, to bolster HBsAg clearance, their results remain pending. Moreover, a study listed on the Australia New Zealand Clinical Trials Registry (http://www.anzctr.org.au/, accessed on 9 July 2023), under the identifier ACTRN12615001133527, highlighted the safety and effectiveness of the PD-1 inhibitor nivolumab for treating HBV DNA-negative CHB patients, refer to Table 1 for details. Most participants exhibited a reduction in HBsAg levels [20]. We eagerly anticipate this study’s potential to pave the way for breakthroughs in HBV treatment, ushering in more effective future treatment strategies.

### 3.2. Cytotoxic T Lymphocyte Antigen-4 (CTLA-4)

#### 3.2.1. CTLA-4 Expression on Normal T Cells

CTLA-4 and PD-1 both belong to the CD28 co-stimulatory molecule family, but they diverge in the timing of their inhibitory impacts on T cells. CTLA-4 manifests its antagonistic effects during the early phase of T cell activation, specifically within lymph nodes. This results in diminished IL-2 production and downregulation of cell cycle proteins, thereby curtailing the T cell immune response and its proliferation. Conversely, PD-1 intervenes by inhibiting T cell activity during the immune response’s later phase, primarily at tissue sites [21]. Upon antigenic exposure, T cells are activated, expressing CTLA-4. This expression modulates the intensity of specific immune responses by orchestrating the production of cytokines, such as TNF-α and IFN-γ, as well as the differentiation, proliferation, contact, and migration of T cells [22]. Furthermore, the immune activity of cytotoxic T cells is also modulated by regulatory cells, known as Treg. CD4CD25Treg, which highly expresses CTLA-4, leads to reduced positive stimulatory signaling in T cells by decreasing CD80/CD86 expression on APCs. Concurrently, it stimulates the release of free PD-L1 from APCs, exerting a dual inhibitory influence on T cell immune responses [23].

#### 3.2.2. CTLA-4 Expression on HBV-Specific T Cells

In patients with chronic HBV infection, there was a notable elevation in the CTLA-4 expression of HBV-specific CD8+ T cells, and this upswing was strongly tied to the viral load. Moreover, these HBV-specific CD8+ T cells exhibiting heightened CTLA-4 expression also displayed increased levels of the pro-apoptotic agent, Bim. This suggests that CTLA-4 might foster the dysfunction of HBV-specific CD8+ T cells via a Bim-dependent apoptotic process [24]. Such CD8+ T cells, with elevated CTLA-4 levels, further lead to compromised organism-specific immune reactions and a prolonged diminishment in antiviral capabilities [25] (Figure 2). Additionally, CTLA-4 single nucleotide polymorphisms (SNPs) have a potential role in the immune regulation of chronic HBV infection. Specifically, the CTLA4 rs231775 SNP exhibits a robust association with HBV infection chronicity and susceptibility to the disease [26].

#### 3.2.3. Blocking CTLA-4 in the Treatment of HBV Infection

Considering CTLA-4′s immunoregulatory function in HBV infection, in vitro studies have shown that blocking CTLA-4 mRNA in human lymphocytes using RNA interference leads to an elevated expression of IFN-γ and IL-2 [27]. In 2011, the pioneering anti-CTLA4 antibody, Ipilimumab, was approved as a cancer immune checkpoint blockade (ICB) therapy [28]. It has since demonstrated efficacy in treating various cancers, including melanoma, non-small cell lung cancer, mesothelioma, prostate cancer, ovarian cancer, and breast cancer.

Two FDA-approved anti-CTLA-4 antibodies, Ipilimumab and tremelimumab, have shown safety and efficacy in treating HBV/HCV infections and associated hepatocellular carcinoma [29,30]. Then, a combination of tremelimumab and durvalumab received approval in the United States for treating unresectable hepatocellular carcinoma in 2022 [31], signifying the clinical transition of combining anti-CTLA-4 and anti-PD-1 antibodies. Nevertheless, considering CTLA-4′s inherent immunoregulatory role, blocking the CTLA-4 pathway comes with certain risks. There is the potential for immune system imbalances, which might trigger the re-emergence of HBV/HCV or even induce autoimmune diseases. Some experts advocate for retaining partial, rather than complete, inhibition of CTLA-4 immune checkpoint functionality to enhance the safety profile of anti-CTLA-4 antibodies. This approach may chart the future trajectory of immune checkpoint inhibitor therapies [32].

### 3.3. T Cell Immunoglobulin and Mucin Structural Domain-3 (Tim-3)

#### 3.3.1. Tim-3 Expression on Normal T Cells

**Figure 3:**
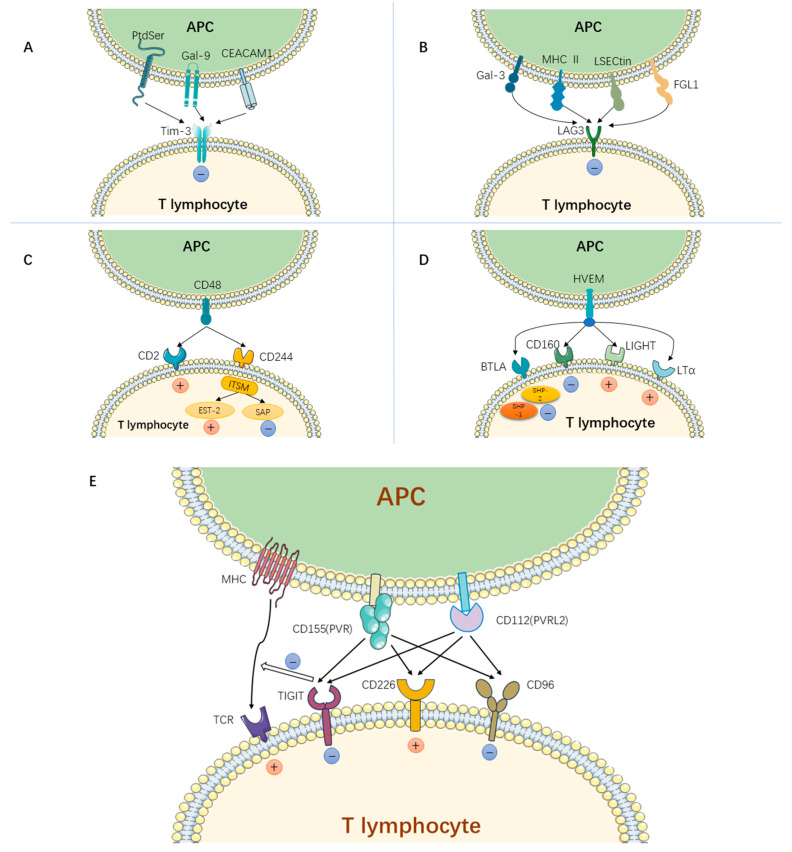
Panel (**A**): Suppressor receptor Tim-3 and its ligand transmit inhibitory signals upon binding; Panel (**B**): Suppressor receptor LAG3 and its ligand transmit signals that inhibit T lymphocyte activation upon binding; Panel (**C**): CD244 binds to its ligand CD48, which is based on the immunoreceptor tyrosine switch motif (ITSM) that transmits stimulatory signals when bound to EST-2 and inhibitory signals when bound to SAP, and CD48 transmits stimulatory signals to T lymphocytes when bound to CD2, thus making CD48 a bidirectional switch; Panel (**D**): In the CD160/BTLA/LIGHT/LTα/HVEM signaling complex, HVEM transmits inhibitory signals when bound to BTLA or CD160, and HVEM transmits inhibitory signals when bound to LIGHT or LTα. Panel (**E**): TIGIT competitively binds CD155 and CD112 with CD226 and transmits inhibitory cell signaling. Also binding to CD155 and CD112 and then transmitting inhibitory signaling is CD96, but TIGIT is dominant among them. In addition to transmitting inhibitory signaling on its own, TIGIT also directly inhibits the TCR signaling pathway. (Created with https://smart.servier.com/, accessed on 12 June 2023).

Recent research indicates that Tim-3 acts as a negative regulator for IFN-γ-producing CD4+ and CD8+ T cells, playing a pivotal role in T cell exhaustion [33]. The ligand for Tim-3, galactose lectin-9 (Gal-9), belongs to the S-type lectin family. It interacts with Tim-3 in a glycosylation-dependent manner, establishing β-glycosidic bonds and consequently triggering Th1 cell apoptosis [34]. While Gal-9 was the inaugural ligand identified for Tim-3, subsequent research has unveiled other ligands, such as phosphatidylserine (PtdSer), high mobility histone B1 (HMGB1) [35], and carcinoembryonic antigen cell adhesion molecule 1 (CEACAM1) [36] (Figure 3). These ligands interact with Tim-3, orchestrating both the innate immune response and T cell function, especially against a backdrop of chronic viral infections and a tumor immune microenvironment.

#### 3.3.2. Tim-3 Expression on HBV-Specific T Cells

In patients with chronic HBV infection, factors like HBV antigenic peptides and γc cytokines collectively induce Tim-3 expression in both CD4+ and CD8+ T cells, which decreases its proliferative capacity and decreases the secretion of antiviral cytokines. This upregulation correlates positively with the severity of liver damage in chronic HBV patients [37,38]. This may be related to the fact that Tim-3-expressing monocytes induce the differentiation of CD4+ T cells to Th17 cells during persistent HBV infection and produce large amounts of IL-17, which activates multiple immune cells to release inflammatory mediators [39]. NK cells expressing Gal-9 can induce dysfunction in Tim-3+ CD8+ T cells through the Gal-9/Tim-3 pathway. In vitro studies have shown that blocking this pathway partially restores the function of CD8+ T cells [40]. This suggests that the Gal-9/Tim-3 pathway may regulate the function of multiple immune cells and impact the immune clearance of HBV.

#### 3.3.3. The Promise of Blocking Tim-3/Tim-3L Pathway in the Treatment of HBV Infection

Immunotherapies that block the Tim-3/Tim-3L signaling pathway have been validated in preclinical models of various solid tumors. Cytotoxic T lymphocytes (CTL) exposed to anti-Tim-3 antibodies displayed heightened antigen specificity. Notably, melanoma-associated antigen-A11 (MAGE-A11)-specific CTLs, when combined with a Tim-3 and PD-1 blockade, demonstrated significantly amplified cytotoxic effects against breast cancer cells [41]. Moreover, combined immunotherapy using NCG gel and anti-Tim-3 antibodies elevated IFN-γ and IL-12p70 levels in hepatocellular carcinoma tissues, facilitating the infiltration of IFN-γ+CD8+ T cells and 41BB+CD8+ T cells. This approach effectively achieved complete remission in a significant proportion (4/7) of cases, prevented lung metastasis from primary hepatocellular carcinoma, and substantially extended survival [42]. Preliminary clinical data suggest enhanced safety and tolerability when combining anti-Tim-3 with anti-PD-1/PD-L1 monoclonal antibodies [43]. Although several studies underscore the potential of blocking the Tim-3/Tim-3L pathway as a treatment for chronic HBV infection [44], relevant clinical trials are notably absent. As research into Tim-3 and its ligands deepens, the Tim-3/Tim-3L pathway is anticipated to emerge as a novel therapeutic avenue for the restoration of anti-HBV immunity.

### 3.4. Lymphocyte Activation Gene 3 (LAG3)

#### 3.4.1. LAG3 Expression on Normal T Cells

Similarly to PD-1 and CTLA-4, naïve T cells do not express LAG3. However, after antigenic stimulation, it becomes expressed on activated CD4+ and CD8+ T cells [45]. Under conditions of prolonged chronic viral infections and sustained antigen exposure, LAG3 remains highly expressed on both CD4+ and CD8+ T cells, contributing to T cell exhaustion [46]. LAG3′s primary canonical ligand is MHC II, with which it demonstrates a higher affinity compared to CD4. By competitively binding to MHC II instead of CD4, LAG3 can convey inhibitory signals through its cytoplasmic domain, thus suppressing T cell activation [47]. In recent times, other potential ligands for LAG3 have been identified. These include galactose lectin-3 (Gal-3), liver-specific adhesion molecule (LSECtin), and fibrinogen-like protein 1 (FGL1) (Figure 3). The binding of these potential ligands to LAG3 showed varying degrees of inhibition of both the activation and proliferation of antigen-specific T cells [48,49,50].

#### 3.4.2. LAG3 Expression on HBV-Specific T Cells

In individuals with chronic HBV infection, there is a notable increase in LAG3 expression on CD4+ T cells. This upregulation is accompanied by a decrease in the secretion of essential cytokines such as IFN-γ, IL-2, and TNF-α. Interestingly, blocking the LAG3 pathway led to a partial restoration of CD4+ T cell function, underscoring LAG3′s inhibitory impact on the immune response during HBV infection [51]. In a study by Ferrando-Martinez et al., the surface inhibitory receptor phenotypes of HBV-specific CD8+ T cells were examined. It was observed that LAG3+CD8+ T cells exhibited a pronounced depletion phenotype, in contrast to LAG3-CD8+ T cells, which showed low to moderate depletion. In addition, varied responses of CD8+ T cells with different phenotypes to inhibitory receptor blockade were observed. Specifically, CD8+ T cells exhibiting the LAG3 phenotype were less receptive to a PD-1 blockade compared to their LAG3 counterparts [17]. This differential response may be attributed to the tiered expression of inhibitory receptors on HBV-specific CD8+ T cells.

#### 3.4.3. The Promise of Blocking LAG3 in the Treatment of HBV Infection

LAG3 has emerged as a pivotal inhibitory receptor in the realm of anticancer immunotherapy, following the lead of PD-1 and CTLA-4. At present, there are 113 clinical trials worldwide focusing on LAG3, spanning a range of conditions, from advanced solid tumors and hematologic diseases to autoimmune disorders and chronic viral infections. The LAG3-targeted therapies that have progressed to clinical trials fall into three categories: anti-LAG3 monoclonal antibodies, anti-LAG3 bispecific antibodies, and LAG3 immunoglobulin (Ig) fusion proteins.

Relatlimab, known as BMS-986016, is a pioneering anti-LAG3 human-derived monoclonal IgG4-κ antibody. It not only marked the clinical debut of anti-LAG3 monoclonal antibodies but also earned the distinction of being the first anti-LAG3 antibody to gain FDA approval [52]. Notably, in these trials, Relatlimab is not used as a standalone treatment. A significant study involving large cohorts of stage III or IV melanoma patients found that pairing anti-LAG3 antibodies with anti-PD-1 antibodies considerably enhanced progression-free survival compared to using anti-PD-1 antibodies alone [53]. Several bispecific antibodies against LAG3 and PD-1 such as MGD013, FS118, IBI323, and ABL501, as well as a LAG3/CTLA-4 bispecific antibody in combination with CTLA-4 (XmAb22841) are currently undergoing preclinical or early clinical studies. LAG-3Ig enhances specific immune responses by promoting antigen cross-presentation to CD8+ T cells and suppressing the proliferation and function of regulatory T cells [54]. IMP321 is the first clinically studied LAG3-targeting molecule and the only one currently being investigated in hepatitis B. In a study with HBsAg-positive participants, those treated with IMP321 exhibited a faster emergence of anti-HBs and a significant rise in CD4+ or CD8+ antigen-specific T cells compared to controls [55]. Targeting LAG3 may be a critical step in restoring anti-HBV immunity in cytotoxic T lymphocytes as well as in developing durable protective immunity.

### 3.5. CD244/2B4 (SLAMF4)

#### 3.5.1. CD244/2B4 Expressed on Normal T Cells

2B4, also referred to as CD244 or SLAMF4, is a receptor that does not bind to MHC. It is a member of the CD150 subgroup within the CD2 receptor family [56]. Its primary ligand, CD48 (or SLAMF2), belongs to the same SLAM family as CD244 and is prevalent across a majority of immune cells. Notably, CD48 has the ability to interact with CD2, which leads to the activation of effector T cells and antigen-presenting cells, classifying it as a stimulatory receptor [57]. The dynamic between CD244 and CD48 is multifaceted, resulting in either stimulatory or inhibitory signals that influence immune responses. The nature of these signals may be determined by the intracellular proteins that CD244 binds to. For instance, when CD244′s ITSM binds with the intracellular SLAM-associated protein (SAP), it sends out stimulatory signals. On the other hand, when it is associated with Ewing sarcoma transcript-2 (EST-2), inhibitory signals are produced [58] (Figure 3).

#### 3.5.2. CD244/2B4 Expression on HBV-Specific T Cells

CD244 demonstrates differential expression in various stages of HBV infection. In patients with chronic HBV infection, the expression level of CD244 on virus-specific CD8+ T cells in the peripheral blood and liver is significantly higher compared to patients with acute infection [59]. This suggests a close association between the expression of CD244 on HBV-specific T cells and the chronicity of HBV infection. Additionally, CD244 on T cells exhibited a parallel relationship with PD-1 expression, and blocking the CD244 or CD48 pathway resulted in the restoration of certain immune functions in T cells. Importantly, this restoration was independent of PD-1 expression [60]. These findings suggest that the expression of CD244 may play an independent role in the chronicity of HBV infection. It is now believed that the possible mechanism is that Lnc-AIFM2-1 acts as an endogenous competitive RNA (ceRNA) for miR-330-3p to regulate the expression of CD244 on CD8+ T cells and contributes to the immune escape of HBV [61]. Studies conducted at the epigenetic level have revealed that examining the gene regulation process in immune cells during HBV clearance can provide valuable insights into the expression of crucial genes that determine the function of immune cells. These studies aim to uncover molecular markers and indicators of genetic immunology that play a role in determining HBV clearance.

#### 3.5.3. The Promise of Blocking CD244/2B4 in the Treatment of HBV Infection

While many studies have shown that increased CD244 expression on CD8+ T cells leads to immunosuppression and contributes to HBV immune evasion, there have been no clinical trials introducing specific blockers. This hesitation stems from a limited understanding of the molecular mechanisms by which the CD244 signaling pathway depletes effector T cell function, coupled with ongoing evaluations of the potential risks and benefits of such a blockade.

### 3.6. CD160

#### 3.6.1. CD160 Expression on Normal T Cells

CD160 is a type I membrane protein that exists in two isoforms: glycosylphosphatidylinositol-anchored (CD160-GPI) and the transmembrane (CD160-TM). CD160 binds to the herpesvirus entry mediator (HVEM), playing a pivotal role in suppressing T cells, activating natural killer cells, and bolstering mucosal immunity [62]. For T lymphocytes, the HVEM, when bound to CD160, sends co-inhibitory signals, resulting in the suppression of CD4+ T cell proliferation and a reduction in IFN-γ secretion [63]. Furthermore, apart from CD160, it binds to several ligands, including BTLA (B and T lymphocyte attenuator), LIGHT (related to lymphotoxin with an inducible expression that competes with herpes simplex virus glycoprotein D for HVEM, a receptor expressed on T lymphocytes), and LTα (lymphotoxin-α) (Figure 3). When LIGHT or LTα binds to HVEM, it sends a co-stimulatory signal, whereas the binding of BTLA or CD160 results in a co-inhibitory signal. As a result, HVEM functions as a two-way switch, either stimulating or inhibiting T cell activation. This intricate network of interactions, encompassing HVEM and its various ligands, establishes a multifunctional CD160/BTLA/LIGHT/LTα/HVEM signaling complex [62,64].

#### 3.6.2. CD160 Expression on HBV-Specific T Cells

In a mouse model studying HBV infection, researchers observed an uptick in CD160 expression on CD8+ T cells. This rise was accompanied by a notable reduction in the release of the inflammatory cytokines IFN-γ and TNF-α. The transcription factor Eomes appears to play a role in modulating the expression of inhibitory receptors on T cells. Notably, Eomes seems to directly foster CD160 expression. Additionally, it indirectly boosts the co-expression of suppressor receptors such as PD-1, LAG-3, and CD160. It achieves this by amplifying the transcriptional abilities of other pivotal transcription factors, namely, NFATc1, Blimp3, and FoxO1, culminating in T cell dysfunction [65].

On the epigenetic front, research highlighted that lncRNA-CD160 expression levels in peripheral blood CD8+ T cells of HBV-infected patients were notably high. Elevated lncRNA-CD160 expression was linked to a decrease in the secretion of IFN-γ and TNF-α by CD8+ T cells. Importantly, the expression of lncRNA-CD160 on CD8+ T cells was significantly curtailed following the suppression of HBV virus replication [66]. CD160 may play an important role in HBV-specific T cell failure, but more studies are needed to confirm the exact mechanism.

#### 3.6.3. The Promise of Blocking CD160 in the Treatment of HBV Infection

While the molecular architecture and ligand interactions of the CD160 protein have been thoroughly examined, its in vivo function and role remain subjects of ongoing research. This is largely due to the intricate interaction dynamics of the CD160/BTLA/LIGHT/LTα/HVEM signaling network. CD160 has been implicated in various conditions, including malignancies, atherosclerosis, malaria, viral infections, autoimmune disorders, and eye diseases [67]. Currently, the anti-CD160-TM antibody (NCT04477876) is undergoing clinical trials to assess its safety and efficacy for patients with inoperable stage III or IV melanoma. However, the outcomes of these trials are yet to be published.

### 3.7. B and T Lymphocyte Attenuator (BTLA)

BTLA, the third member of the CD28 superfamily, was identified after PD-1 and CTLA-4. It shares structural similarities with PD-1 and CTLA-4 proteins. Predominantly found in lymphocytes, BTLA expression is more pronounced in CD4+ T cells compared to CD8+ T cells [68]. When BTLA interacts with its ligand, HVEM, it undergoes tyrosine phosphorylation, leading to the recruitment of phosphatase SHP1/2 to its ITIM motif. This interaction dampens TCR signaling and sends inhibitory signals within the cell [69]. BTLA has a key regulatory role in a variety of inflammatory and infectious immune processes and is a part of the intricate CD160/BTLA/LIGHT/LTα/HVEM signaling network [62] (Figure 3).

BTLA expression on T cells may be tissue-specific, the research indicates a heightened expression of BTLA on HBV-specific T cells in the livers of individuals with chronic HBV infection. This expression facilitates IL-10 secretion, diminishing the effector capabilities of HBV-specific CD8+ T cells and subsequently suppressing their antiviral responses [70]. A notable functional impairment is seen in intrahepatic T cells co-expressing BTLA and PD-1. However, interrupting the BTLA pathway can rejuvenate the proliferation and functionality of certain intrahepatic and peripheral T cells [71]. Currently, anti-BTLA monoclonal antibodies like JS004, TAB004, and HFB200603 are under investigation for their safety and potential in treating advanced malignancies. There is anticipation that therapies targeting BTLA will soon extend to a range of chronic infectious diseases, including HBV.

### 3.8. T Cell Immunoreceptor with Ig and ITIM Domains (TIGIT)

TIGIT is a prominent receptor expressed in a variety of immune cells, including activated T cells, memory T cells, Treg cells, and follicular T helper (Tfh) cells. Recognized for its inhibitory role in immune cells, TIGIT can effectively suppress T cell activation through various mechanisms [72]. These include binding to its ligands, targeting molecules within the TCR signaling pathway, and participating in the downregulation of TCR complex components (Figure 3). Furthermore, TIGIT plays a role in promoting the expression of certain molecules, including anti-apoptotic ones like Bcl-xL, and cytokines such as IL-2, IL-7, and IL-15, which help in sustaining T cell survival [73].

In a study involving patients with chronic HBV infection, the elevated expression of TIGIT was observed on T cells, particularly on effector T cell subsets compared to naïve T cells and memory T cell subsets. CD8+ T cells with high levels of TIGIT expression exhibited significantly reduced the secretion of cytokines such as IFN-γ, IL-2, and TNF-α. Blocking the TIGIT signaling pathway was found to restore the function of certain T cells [74]. Moreover, TIGIT has a multifaceted role in the progression of HBV-associated HCC. While it fosters hepatic immune tolerance and induces CD8+ T cell dysfunction in the precancerous stage, its enhanced expression during chronic HBV infection might favor tumor growth by curbing the anti-tumor responses of effector T cells [75]. TIGIT has diverse roles in HBV-associated hepatocellular carcinoma (HCC) development and progression. The use of checkpoint inhibitors targeting TIGIT at different disease stages can yield contrasting outcomes. Therefore, the precise timing and control of anti-TIGIT monoclonal antibody treatment are crucial when applied to HBV-infected patients to ensure optimal results.

## 4. Conclusions

In chronic HBV infection, HBV-specific CD8+ T cells exhibit structured expression of inhibitory receptors [17]. T cell exhaustion arises from diverse molecular pathways active in varying immune response phases [9,21]. Given the limited effectiveness of single immune checkpoint inhibitors in restoring T cell function, the integration of multiple inhibitors and the development of composite inhibitors are crucial for advancing clinical trials and treatment strategies in chronic HBV infections. However, a complete blockade of these pathways may disrupt immune balance and risk HBV relapse. When combining immune checkpoint inhibitors, a careful balance must be struck to preserve some checkpoint functions, enhancing safety [32]. Further studies are essential to understand the interplay between different checkpoint pathways and to determine the most effective combinations for chronic HBV patients.

In the current therapeutic landscape, the evolution of nucleotide analogs targeting HBV DNA replication has progressed from initial successes to a state of maturity. Concurrently, the clinical deployment of interferon, aimed primarily at facilitating the clearance of HBsAg, has become widespread. However, the efficacy of interferon in achieving a functional cure remains an area necessitating further optimization. Research on T cell suppressor receptors indicates that targeting receptors that do not bolster the body’s immune response and pairing immunotherapy with antiviral treatments may offer a promising path to a functional cure for individuals with chronic HBV infection. As a result, future research will prioritize devising combination therapeutic approaches to address chronic HBV infection more effectively.

## Figures and Tables

**Table 1:** Clinical trials of T lymphocyte-associated inhibitory receptors in HBV infection.

Immune Checkpoint Pathway	NCT Number	Phase	Study Start Date	Status	Inclusion Criteria	Mechanism	Drug	Primary Outcome Measures	Results	Locations
PD-1/PD-L	NCT04638439	Phase 2	December 2021	Recruiting	CHB patients; HBsAg < 1500 IU/mL; HBV DNA (−)	Anti-PD-1	P1101 Nivolumab Entecavir	Safety	N/A	Taiwan, China
	NCT05769816	Phase 4	June 2023	Not yet recruiting	CHB patients	Anti-PD-1	Anti-PD-1 antibody NAs	Serum HBsAg level	N/A	Chongqing, China
	NCT05771402	Phase 4	June 2023	Not yet recruiting	CHB patients	Anti-PD-1	Anti-PD-1 antibody NAs Peg-IFNα	Serum HBsAg level	N/A	Chongqing, China
	NCT05275023	Phase 2	June 2022	Active, not recruiting	CHB patients; LSM ≤ 9 KPa/ metavir F0-F2	Anti-PD-1	JNJ-73763989 PD-1 Inhibitor NAs	HBsAg seroclearance	N/A	Toronto, Canada
	NCT04133259	Phase 2	December 2019	Unknown	CHB patients; HBsAg > 100 IU/mL; HBV DNA < 2000 IU/mL	Anti-PD-1	HLX10 NAs	Levels of HBsAg decline	N/A	Taiwan, China
	NCT05242445	Phase 1	April 2022	Active, not recruiting	CHB patients; LSM ≤ 9 KPa/ metavir F0-F2	Anti-PD-1	Cetrelimab (JNJ-63723283) Placebo	Pharmacokinetics, Pharmacodynamics, and safety of single doses of Cetrelimab (JNJ 63723283),	N/A	Belgium; France; Germany; Poland; Spain
	NCT04294498	Phase 2	November 2020	Recruiting	HBeAg (−); HBsAg (+); HBV DNA ≥ 2000 IU/mL; Barcelona Clinic Liver Cancer (BCLC) Stage C disease or BCLC Stage B disease not amenable to locoregional therapy (Advanced Hepatocellular Carcinoma)	Anti-PD-1	Durvalumab	The rate of HBV reactivation during durvalumab treatment	N/A	Taiwan, China
	NCT04044651	Phase 3 Phase 2	October 2019	Withdrawn	HBsAg (+); BCLC (C stage)	Anti-PD-1	Nivolumab Lenvatinib	Overall survival (OS). Time frame: 18 months	N/A	Guangdong, China
	NCT04465890	Phase 2	July 2020	Recruiting	HBV DNA (−); HBeAg (−); HBsAg ≤ 10,000 IU/mL	Anti-PD-L1	PD-L1 Antibody (ASC22)	Levels of HBsAg decline	N/A	Beijing, China
	NCT04225715	Phase 2	July 2020	Recruiting	HBsAg (+) ≥ 6 months; HBV DNA below the lower LLOQ or <20 IU/mL for >6 months	Anti-PD-L1	NUCs CpAM (RO7049389) TLR7 (RO7020531) siRNA (RO7445482) PEG-IFN PD-L1 LNA (RO7191863)	HBsAg seroclearance	N/A	54 study locations including United States, Canada et al.
	NCT04680598	N/A	December 2020	Recruiting	HBsAg (+); HCC (for EASL)	Anti-PD-1/anti-PD-L1	ICI (including PD-1 inhibitor or PD-L1 inhibitor); NAs	HBV reactivation rate	N/A	Guangdong, China
	NCT03419481	Phase 2	April 2018	Recruiting	HBsAg (+); HBV DNA < 100 IU/mL; HCC (for AASLD)	Anti-PD-L1	pembrolizumab	Response rate (RR) according to RECIST1.1. Time frame: 2 years	N/A	Hong Kong, China
LAG3	NCT00354861	Phase 1	May 2005	Completed	Healthy	hLAG-3Ig	IMP321 (AG-3)	Safety and tolerability profiles. Time frame: 3 months	IMP321 was very well tolerated	Paris, France

**Table 2:** Search form and results of each database.

PubMed	Search query: (“Hepatitis B”MeSH Terms OR “HBV”All Fields) AND (“Costimulatory and Inhibitory T-Cell Receptors”MeSH Terms OR (“inhibitory”All Fields AND (“cell cycle checkpoints”MeSH Terms OR (“cell”All Fields AND “cycle”All Fields AND “checkpoints”All Fields) OR “cell cycle checkpoints”All Fields OR “checkpoint”All Fields OR “checkpoints”All Fields)) OR ((“immune”All Fields OR “immuned”All Fields OR “immunes”All Fields OR “immunisation”All Fields OR “vaccination”MeSH Terms OR “vaccination”All Fields OR “immunization”All Fields OR “immunization”MeSH Terms OR “immunisations”All Fields OR “immunizations”All Fields OR “immunise”All Fields OR “immunised”All Fields OR “immuniser”All Fields OR “immunisers”All Fields OR “immunising”All Fields OR “immunities”All Fields OR “immunity”MeSH Terms OR “immunity”All Fields OR “immunization s”All Fields OR “immunize”All Fields OR “immunized”All Fields OR “immunizer”All Fields OR “immunizers”All Fields OR “immunizes”All Fields OR “immunizing”All Fields) AND (“cell cycle checkpoints”MeSH Terms OR (“cell”All Fields AND “cycle”All Fields AND “checkpoints”All Fields) OR “cell cycle checkpoints”All Fields OR “checkpoint”All Fields OR “checkpoints”All Fields)) OR (“PD-1”All Fields OR (“ctla 4 antigen”MeSH Terms OR (“ctla 4”All Fields AND “antigen”All Fields) OR “ctla 4 antigen”All Fields OR “ctla 4”All Fields) OR “LAG3”All Fields OR “Tim-3”All Fields OR “cd244”All Fields OR “CD160”All Fields OR “TIGIT”All Fields OR “BTLA”All Fields)) AND (“T-Lymphocytes”MeSH Terms OR (“T-Lymphocytes”MeSH Terms OR “T-Lymphocytes”All Fields OR “t cell”All Fields))
	Results: 396
Web of Science	((hepatitis B (Topic) or HBV (Topic) and Preprint Citation Index (Exclude—Database)) AND inhibitory receptors (Topic) or inhibitory checkpoints (Topic) or immunization checkpoints (Topic) and Preprint Citation Index (Exclude—Database)) AND T lymphocyte (Topic) or T cell (Topic) and Preprint Citation Index (Exclude—Database) and Preprint Citation Index (Exclude—Database)
	Result: 492
Scopus	(((ALL(hepatitis B) OR ALL(HBV))) AND ((ALL(inhibitory AND receptors) OR ALL(inhibitory AND checkpoints) OR ALL(immunization AND checkpoints))) AND ((ALL(T lymphocyte) OR ALL(T cell))) AND (LIMIT-TO (LANGUAGE,”English”)) AND (LIMIT-TO (DOCTYPE,”ar”)) AND (LIMIT-TO (EXACTKEYWORD,”Hepatitis B”) OR LIMIT-TO (EXACTKEYWORD,”T-Lymphocytes”) OR LIMIT-TO (EXACTKEYWORD,”Hepatitis B Virus”) OR LIMIT-TO (EXACTKEYWORD,”T Lymphocyte”)))
	Results: 3310

## Data Availability

The data used to support the findings are available from the corresponding author upon request.

## Abbreviations

HBeAg, hepatitis B e antigen; MHC, major histocompatibility complex; IL-2, Interleukin-2; IFN-γ, interferon-γ; TNF, tumor necrosis factor; IL-10, Interleukin-10; TGF-β, transforming growth factor-β; AHB, acute hepatitis B; CHB, chronic hepatitis B; HBV-HCC, HBV-related hepatocellular carcinoma; HBV-ACLF, hepatitis B virus-related acute-on-chronic liver failure; HIV, human immunodeficiency virus; HCV, hepatitis C virus; HDV, hepatitis D virus; CMV, cytomegalovirus; ITIM, immunoreceptor tyrosine-based inhibitory motif; ITSM, immunoreceptor tyrosine-based switch motifs.
